# Minding the “T”s beyond the “B”s: Shaping vaccines for future pandemics

**DOI:** 10.1371/journal.pbio.3002351

**Published:** 2023-11-07

**Authors:** Shirin Kalimuddin, Eng Eong Ooi

**Affiliations:** 1 Department of Infectious Diseases, Singapore General Hospital, Singapore; 2 Programme in Emerging Infectious Diseases, Duke-NUS Medical School, Singapore; 3 Viral Research and Experimental Medicine Centre, SingHealth Duke-NUS Academic Medical Centre, Singapore; 4 Department of Clinical Research, Singapore General Hospital, Singapore; 5 Saw Swee Hock School of Public Health, National University of Singapore, Singapore

## Abstract

In this Perspective, Eng Eong Ooi and colleagues argue that a failure to integrate T cell immunity as a determinant of vaccine efficacy could curtail the development of newer vaccines to help us prepare for future pandemics.

## Teaser

The COVID-19 pandemic has accelerated the development of vaccines for viral infections. However, a failure to integrate T cell immunity as a determinant of vaccine efficacy could curtail advancement of newer vaccines for pandemic preparedness.

An undeniable silver lining of the Coronavirus Disease 2019 (COVID-19) pandemic has been the unprecedented pace at which vaccine development and testing were conducted. Vaccination was arguably the main intervention that brought the pandemic under control, allowing lockdowns to be lifted and borders to reopen. This experience now positions rapid vaccine development as a key element of pandemic preparedness, with the Coalition for Epidemic Preparedness Innovations (CEPI) issuing a challenge to respond to the next pandemic with a safe and effective vaccine within 100 days [1].

To respond even more rapidly than we were able to for COVID-19, advances in vaccine technology will be needed. Besides increasing the speed and yield of production, approaches could also be taken to increase vaccine potency and product stability, some of which are already actively being explored. For example, one avenue for enhancing the stability and delivery efficiency of mRNA vaccines is to alter RNA and RNA-packaging nanoparticle chemistry, such as by adding nucleotide modifications or altering the lipid composition of the nanoparticles. Reductions in dosing requirements would reduce costs, thereby increasing access, and enable each batch of vaccine to protect a larger population. Similarly, genetic modification of currently employed viral vectors, such as adenoviruses and vaccinia viruses, or even the development of new viral vectors, could improve the immunogenicity of such vaccines. Research is also underway to develop newer and more potent adjuvants to augment the potency of protein-based and inactivated viral vaccines.

The advancement of vaccine technology is exciting. However, there is a danger that such advancements could be curtailed if we fail to consider the effects of the vaccine on immunity as a whole. In the case of COVID-19 vaccines, it was fortuitous that the spike protein of Severe Acute Respiratory Syndrome Coronavirus 2 (SARS-CoV-2) contained both B cell and T cell epitopes, resulting in a robust humoral immune response (antibody production) and cellular immune response (virus-specific CD4^+^ (helper) and CD8^+^ (killer) T cells) to vaccination. Indeed, the body of evidence supporting cellular immunity as a key protector against COVID-19 is now solid. For example, the onset of mRNA vaccine–induced protection, which occurred as early as 12 days after vaccination, was temporally related to the appearance of spike-reactive T cells; neutralizing antibodies against SARS-CoV-2 were undetectable at this time point [2]. Moreover, as vaccine-induced neutralizing antibody titers waned, preservation of vaccine-induced T cell responses, which were also less susceptible to escape mutations than neutralizing antibodies, protected against severe COVID-19 even as SARS-CoV-2 evolved [3]. Similarly, adenovirus-vectored vaccines were able to elicit robust CD4^+^ and CD8^+^ T cell responses, translating to high levels of vaccine efficacy [4] that contrasted with inactivated virus vaccines, which were not able to induce a CD8^+^ T cell response [5].

In addition to SARS-CoV-2, T cell immunity is also important for other viral infections. Transcriptomic profiling of individuals infected with dengue revealed that those who were viremic but asymptomatic had increased T cell activation, whereas those with symptomatic dengue had increased plasmablast (antibody-producing B cell) differentiation [6]. Similarly, the lack of sustainable efficacy of the live-attenuated dengue vaccine, Dengvaxia, has been attributed, at least in part, to the lack of a vaccine-induced, dengue-specific T cell response, despite the vaccine generating high titers of neutralizing antibodies [7]. These and other observations drive home the notion that vaccines that generate both humoral and cellular immunity are needed for sustainable protection against acute viral diseases.

The use of neutralizing antibody titer as a primary selection factor for vaccine development would not be problematic if neutralizing antibody titers correlated directly with T cell responses to vaccination. However, the dynamics of antibody production and T cell development are decoupled. For example, patients with severe COVID-19 show high antibody titers but reduced T cell responses to SARS-CoV-2 infection [8]. T cell responses can be produced by subclinical SARS-CoV-2 infection without seroconversion (i.e., without developing a detectable antibody response) [9]. Moreover, a systems vaccinology study showed that the innate immune responses to SARS-CoV-2 mRNA vaccination that shaped the antibody response were distinct from those that correlated with T cell responses [10]. Given that distinct host response pathways shape antibody and T cell responses, neutralizing antibody titers cannot inform on the magnitude and quality of the T cell response to vaccination. It is thus plausible that by designing vaccines to elicit host responses in antigen-presenting cells that favor B cells, we could be adversely affecting the development of T cell responses.

Although live-attenuated virus vaccines are most suited to induce robust antibody and T cell responses (the latter against the entire viral proteome), the use of this vaccine platform in a pandemic setting is unfortunately challenging due to biosafety concerns. Moreover, such vaccines cannot be administered to high-risk groups such as immunocompromised or pregnant individuals, which restricts their potential for large-scale deployment during a pandemic. It is thus imperative that other vaccine platforms be refined to improve their ability to stimulate a strong cellular immune response. For example, we need to develop adjuvants that promote the cross-presentation of viral proteins to CD8^+^ T cells by MHC class I molecules; such cross-presentation will be crucial for the development of protein-based and inactivated viral vaccines, as these vaccines contain antigens that are exogenously taken up by antigen-presenting cells and would otherwise mostly be presented on MHC class II molecules to stimulate CD4^+^ T cells.

For T cell immunity to shape vaccine development, standardized and scalable T cell assays are urgently needed. Unlike antibody assays, where standardized international units and commercial kits exist, T cell assessments remain operator dependent and low throughput. Fortunately, another silver lining of the COVID-19 pandemic has been the advent of more user-friendly T cell assays. One example is a recently developed rapid cytokine-release assay to assess SARS-CoV-2–specific T cell responses, an assay that requires less than 1 ml of whole blood and has shown good sensitivity and specificity when compared to conventional T cell assays [11]. Further development of such simple-to-use assays could standardize T cell measurements not only to guide vaccine development, but also to expand the parameters used to define correlates of protection beyond the current exclusive reliance on antibody measurements.

The COVID-19 pandemic has ushered in a new era of vaccine advancement, showcasing the potential of both mRNA vaccines as well as encouraging further development of other diverse vaccine platforms. As we advance vaccine platforms to meet future challenges, it will be crucial to consider vaccine immunogenicity beyond the context of just neutralizing antibodies. Lessons from the COVID-19 pandemic (and from other viral infections) have highlighted the importance of T cell immunity in providing sustained protection, especially against severe disease. Shaping vaccines to produce optimal humoral and cellular immunity will be critical in ensuring we are able to respond effectively to future viral pandemics.

## Abbreviations

CEPI: Coalition for Epidemic Preparedness Innovations
COVID-19: Coronavirus Disease 2019
SARS-CoV-2: Severe Acute Respiratory Syndrome Coronavirus 2

## References

[1] Usher AD. CEPI launches 100-day vaccine “moonshot”. Lancet. 2022;399:1107–1108. doi: 10.1016/S0140-6736(22)00513-x 35305730 PMC8930004

[2] Kalimuddin S, Tham CYL, Qui M, de Alwis R, Sim JXY, Lim JME, et al. Early T cell and binding antibody responses are associated with COVID-19 RNA vaccine efficacy onset. Med. 2021;2:682–688.e4. doi: 10.1016/j.medj.2021.04.003 33851143 PMC8030737

[3] Vardhana S, Baldo L, Morice WG, Wherry EJ. Understanding T cell responses to COVID-19 is essential for informing public health strategies. Sci Immunol. 2022;7:eabo1303. doi: 10.1126/sciimmunol.abo1303 35324269 PMC10344642

[4] Zhang Z, Mateus J, Coelho CH, Dan JM, Moderbacher CR, Gálvez RI, et al. Humoral and cellular immune memory to four COVID-19 vaccines. Cell. 2022;185:2434–2451.e17. doi: 10.1016/j.cell.2022.05.022 35764089 PMC9135677

[5] Premikha M, Chiew CJ, Wei WE, Leo YS, Ong B, Lye DC, et al. Comparative Effectiveness of mRNA and Inactivated Whole-Virus Vaccines Against Coronavirus Disease 2019 Infection and Severe Disease in Singapore. Clin Infect Dis. 2022;75:1442–1445. doi: 10.1093/cid/ciac288 35412612 PMC9047219

[6] Simon-Lorière E, Duong V, Tawfik A, Ung S, Ly S, Casadémont I, et al. Increased adaptive immune responses and proper feedback regulation protect against clinical dengue. Sci Transl Med. 2017;9:eaal5088. doi: 10.1126/scitranslmed.aal5088 28855396

[7] Ooi EE, Kalimuddin S. Insights into dengue immunity from vaccine trials. Sci Transl Med. 2023;15:eadh3067. doi: 10.1126/scitranslmed.adh3067 37585506

[8] Tan AT, Linster M, Tan CW, Le Bert N, Chia WN, Kunasegaran K, et al. Early induction of functional SARS-CoV-2-specific T cells associates with rapid viral clearance and mild disease in COVID-19 patients. Cell Rep. 2021;34:108728. doi: 10.1016/j.celrep.2021.108728 33516277 PMC7826084

[9] Swadling L, Diniz MO, Schmidt NM, Amin OE, Chandran A, Shaw E, et al. Pre-existing polymerase-specific T cells expand in abortive seronegative SARS-CoV-2. Nature. 2022;601:110–117. doi: 10.1038/s41586-021-04186-8 34758478 PMC8732273

[10] Arunachalam PS, Scott MKD, Hagan T, Li C, Feng Y, Wimmers F, et al. Systems vaccinology of the BNT162b2 mRNA vaccine in humans. Nature. 2021;596:410–416. doi: 10.1038/s41586-021-03791-x 34252919 PMC8761119

[11] Tan AT, Lim JM, Le Bert N, Kunasegaran K, Chia A, Qui MD, et al. Rapid measurement of SARS-CoV-2 spike T cells in whole blood from vaccinated and naturally infected individuals. J Clin Invest. 2021;131:e152379. doi: 10.1172/JCI152379 34623327 PMC8409582

